# Beyond the Variants: Mutational Patterns in Next-Generation Sequencing Data for Cancer Precision Medicine

**DOI:** 10.3389/fcell.2020.00370

**Published:** 2020-05-19

**Authors:** Megan Parilla, Lauren L. Ritterhouse

**Affiliations:** ^1^Department of Pathology, University of Chicago Medicine, Chicago, IL, United States; ^2^Center for Integrated Diagnostics, Department of Pathology, Massachusetts General Hospital and Harvard Medical School, Boston, MA, United States

**Keywords:** mutational signature, tumor mutational burden, microsatellite instability, homologous recombination deficiency, mutational pattern

## Abstract

Massively parallel sequencing, also referred to as “next-generation sequencing” (NGS) provides not only information about simple, single nucleotide alterations, but it can also provide information on complex variations, such as insertions and deletions, copy number alterations, and structural variants. In addition to identifying individual alterations, genome-wide biomarkers can be discerned from somatic cancer NGS data, broadly termed mutational patterns and signatures. This review will focus on several of the most common genome-wide biomarkers such as tumor mutational burden, microsatellite instability, homologous recombination deficiency, and mutational signatures.

## Introduction

For decades, scientists have known that cancer is the result of changes to the genomes of cells. Since 1977 DNA sequencing technology has been one tool in the arsenal of scientists to detect these genetic alterations that lead to malignancy (Sanger et al., 1977). Unfortunately, initial Sanger sequencing was time consuming and expensive, and it was not until 2005, when massively parallel sequencing became commercially available, that large scale, cost-effective and efficient sequencing could be done in clinical settings (Mardis, 2011). At the same time, directed therapy for cancer had become increasingly common as more drugs were designed to target specific gene changes in malignancy. This combination of increased clinical utility and technical practicality has placed new emphasis on discovering mutations and chromosomal structure rearrangements, and thus sequencing portions of malignant genomes has become a part of routine clinical practice for some tumor types (Sequist et al., 2011; Nagarajan et al., 2017; Zehir et al., 2017).

Massively parallel sequencing, also referred to as “next-generation sequencing” (NGS) provides not only information about simple, single nucleotide alterations, but it can also provide information on complex variations, such as insertions and deletions, copy number alterations and structural variants. Sequencing can be done on neoplastic tissues, looking for acquired, somatic changes, or it can be done on non-neoplastic tissue to detect germline, inherited differences. Sequencing both patient neoplastic and non-neoplastic tissue together can assist in the interpretation of somatic variations.

Single nucleotide variants are the simplest alteration detected by sequencing technology, and the most common alteration identified, where one nucleotide (A, T, G, or C) is exchanged for an alternate nucleotide. These variations include a range of effects from pathogenic changes to commonly inherited benign polymorphisms. An insertion is the result of additional nucleotides inserted into genetic material, where they typically do not reside, without a one-for-one nucleotide exchange. A genetic deletion is the opposite of an insertion, where nucleotides are lost from the genome, and the total number of nucleotides in decreased. Insertion–deletion variants (indels) are combinations of nucleotide losses and gains. Indels are identified by NGS easily when they are small, but with more difficulty when they are larger. Larger and more complex variations can be identified after post-processing of NGS data with an assembly based realigner (Mose et al., 2014; Au et al., 2017).

Humans are diploid with two copies of each gene. Often, in cancer this copy number is altered. A gene, or large area of the genome can be duplicated, resulting in more copies of a particular gene or genes or alternately, genetic material can be lost such that there is less than the expected number of copies of a gene. These duplications and deletions can be identified in next generation data through analyzing the sequence depth obtained for a locus. The number of reads are then normalized to a pooled reference to determine whether an area is truly amplified or lost. The normalization of reads to a pooled reference assists in controlling for alternate factors that would be expected to change read depth, for instance GC content, the size of the target area of interest or repetitive areas (Talevich et al., 2016).

Although classically detected with cytogenetic methods, chromosomal translocation can also be detected via NGS. A translocation is when a portion of the genome is relocated to an alternate space where it fuses to existing genetic material. Gene fusions, where two different genes become one functional product, is occasionally the result of translocation, but can also occur in the context of large deletions or other complex structural alterations. Using slightly different techniques, NGS can also identify translocations or gene fusions, either through paired-end read alignments or, in the case of some gene fusions, utilizing cDNA obtained after reverse transcription of mRNA.

Each of these above described changes, that can be seen in cancer and detected by NGS, represent events taken in isolation. They answer the primary questions clinicians want to know such as: “Is *TP53* mutated?” or “Is *MET* amplified?” Each of these questions could also be answered with different methodologies, in the above example, using PCR to examine *TP53* or through FISH analysis of *MET*. However, one of the major benefits to NGS technology is that through the analysis of huge swaths of the genome, patterns can be seen in secondary analysis of sequencing data. These patterns can provide additional information: they provide clues to the origin of the tumor, suggestions of past environmental exposures, insights into specific biologic defects in DNA repair, and even expose underlying weaknesses in the tumor themselves, with subsequent recommendations for optimal treatment (Alexandrov et al., 2013, 2020). This chapter will focus on several genome-wide metrics or biomarkers that can be discerned somatic cancer NGS data, broadly termed mutational patterns and signatures.

## Tumor Mutational Burden

**FIGURE 1:**
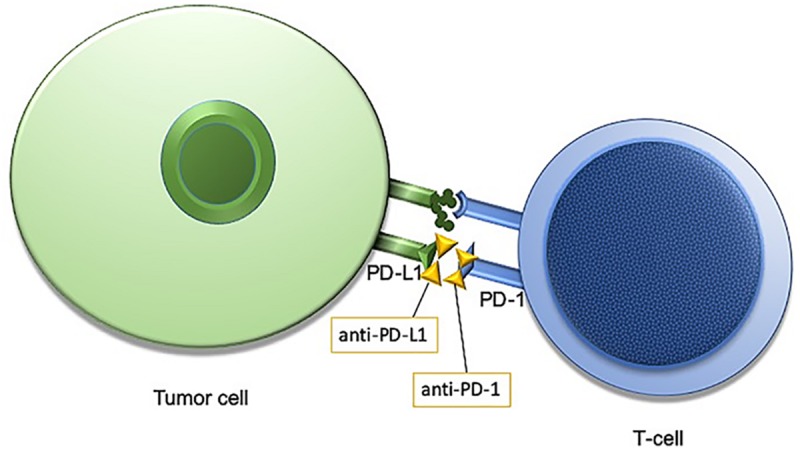
Programmed cell death protein 1/programmed cell death protein ligand1 (PD-1/PD-L1) interaction is an immune checkpoint targeted by anti-PD-1/PD-L1 immunotherapies.

The human immune system is naturally primed to recognize tumor cells as foreign and destroy them. One way for cancer cells to evade the innate T-cell response is through increased activity of a transmembrane protein called programmed death ligand 1, PD-L1. In healthy tissues PD-L1 is expressed on the surface of cells where it interacts with the T-cell receptor, programmed cell death protein 1, PD-1 to promote self-tolerance and suppress the T-cells from killing normal tissues. In cancer, despite expression of MHC I-associated molecules with “foreign cancer” antigens, PD-L1 found on these cells continue to suppress T-cells through PD-L1: PD-1 interactions, and some tumor cells may even increase the expression of PD-L1 on the cell surface to enhance this immune suppression (Friss et al., 1989). Antibodies designed against PD-1 and PD-L1 have demonstrated efficacy for a subset of patients within several different tumor types (Figure 1). Notably, not all patients respond to this treatment, and the response rate is highly dependent upon tumor type, with melanoma and non-small-cell lung cancer (NSCLC) generally exhibiting favorable responses (Brahmer et al., 2012; Topalian et al., 2012). Even within NSCLC, however, only a subset of those patients will respond to immunotherapy. Therefore, identifying a biomarker that is both sensitive and specific for tumors which will respond to immune checkpoint inhibition (ICI) is of great priority.

Expression of PD-L1 as assessed by immunohistochemistry was the initial candidate biomarker evaluated for predicting response to ICI (Topalian et al., 2012; Taube et al., 2014). The expression of PD-L1 on tumor cells has been used in several clinical trials and cohort studies with positivity being associated with favorable response to ICI; however, the sensitivity and specificity of the biomarker is far from perfect (Hellmann et al., 2017; Garon et al., 2019). As such, the interest in identifying an alternative or perhaps complementary biomarker to PD-L1 expression has led to the exploration of tumor mutational burden (TMB) to be one such candidate.

Whole exome sequencing (WES) was performed on NSCLC samples treated with anti-PD-1 therapy who either clinically responded or did not respond to immunotherapy. Therapy response was associated with the number of mutations identified by WES (Rizvi et al., 2015). This simple accounting of mutations across the genome in a tumor was termed TMB and is considered an emerging biomarker for the probability of effective treatment by immunotherapy on numerous tumor types. Interestingly, melanoma and NSCLC have considerably higher median TMB compared to other malignancies, such as hematopoietic, pediatric, or soft tissue tumors, paralleling the superior responses to immunotherapy seen in these tumor types (Reck et al., 2016; Larkin et al., 2019).

The proposed mechanism behind TMB’s utility as a biomarker for immunotherapy is related to increased neoantigen production (Alexandrov et al., 2013; Lawrence et al., 2013; Chalmers et al., 2017; Chen et al., 2017). Tumors with the highest genomic mutational burden will translate more altered protein products which look unlike native peptides. Increased non-native antigens, or neoantigens, expressed by a cell facilitates immune system recognition of the tumor. The inhibition of PD-L1 and PD-1 is a necessary step for immune-modulated destruction, and is facilitated if the tumor expresses an abundance of recognizable neoantigens.

As the clinical utility of TMB was initially described using WES, WES became the gold-standard for quantifying TMB. Unfortunately, due to the lack of routine implementation of WES in clinical practice, implementing tumor-normal WES for routine biomarker testing for immunotherapy is not a practical option. Therefore, in clinical molecular diagnostic testing the aim has shifted to providing accurate TMB estimation with targeted NGS panels, often with tumor-only data. While the size of the targeted panel required for accurate TMB estimation is not well-defined currently, it is generally thought that panels with coverage of at least 1.0 Mb are needed (Allgauer et al., 2018; Buchhalter et al., 2019; Endris et al., 2019; Stenzinger et al., 2019). Smaller panels will have lower correlation to the gold standard, in particular when assessing tumors with low overall mutational burden (Chalmers et al., 2017; Buchhalter et al., 2019). Furthermore, there are many additional technical details for calculating TMB for targeted NGS panels that remain to be fully elucidated, such as the following: what types of variants should be included (missense, indels, etc.), inclusion of coding or non-coding territory, cut-offs used to determine “high” TMB, and best practices for filtering out germline variants to name a few. There are several multi-institutional efforts ongoing that are attempting to create laboratory standards for testing and reporting TMB to harmonize efforts across the world, including initiatives by both the Friends of Cancer Research as well as the Qualitätssicherungs-Initiative Pathologie (QUIP). The preliminary joint report by both Friends of Cancer Research and QUIP provide an excellent overview of many of the technical issues that these standardization initiatives aim to address (Stenzinger et al., 2019). The main issues that their preliminary findings highlight is the importance of targeted gene panel size (at least 1 Mb), panel composition, and bioinformatic pipeline.

In additional to technical considerations for calculating TMB, there are also many questions remaining as to the clinical utility and validity of this new biomarker, as the data from trials is contradictory. In particular, there is a lack of data supporting the association between a high TMB and an improved overall survival following ICI (Planchard et al., 2018). While TMB does not seem to predict outcomes in patients receiving combined ICI and chemotherapy (Paz-Ares et al., 2019), it does seem to be a reliable biomarker of response in patients receiving anti-PD-1 monotherapy in both the first and second lines of therapy (Herbst et al., 2019). While TMB is considered a promising emerging biomarker, further work remains to fully establish both the clinical and technical validity of this genome wide biomarker for its pan-tumor use in predicting clinical response to ICI.

## Mutational Signatures

**FIGURE 2:**
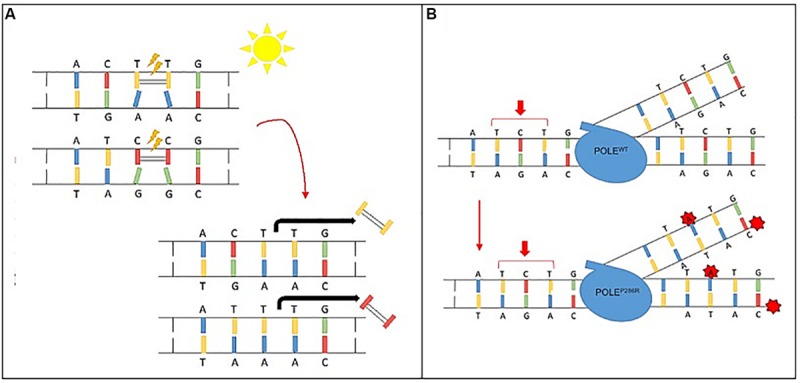
Mutational signature mechanisms. **(A)** Ultraviolet Signature 7 is associated with large numbers of CC > TT dinucleotide mutations at dipyrimidines, as well as a strong transcriptional strand-bias indicating that mutations occur at pyrimidines by formation of pyrimidine–pyrimidine photodimers and that these mutations are being repaired by transcription-coupled nucleotide excision repair. **(B)** POLE Signature 10 exhibits strand bias for C > A mutations at TpCpT context and T > G mutations at TpTpT context. The mutational process underlying this signature is altered activity of the error-prone polymerase POLE due to mutations occurring within the exonuclease domain.

Certain mutagens cause specific genetic alterations to occur more frequently than others; this propensity for one mutational pattern as compared to others is referred to as a “mutational signature.” Mathematical models can plot the total identified mutations seen in a particular tumor and compare to reference data. Such mutational signatures can include both single base substitutions (SBS), doublet base substitutions (DBS), and small insertion and deletions and are detailed in the Catalogue of Somatic Mutations in Cancer (COSMIC) (Tate et al., 2019; Alexandrov et al., 2020). Over 40 SBS signatures have been described with each mutational signature characterized using the six substitution subtypes: C > A, C > G, C > T, T > A, T > C, and T > G. In addition to cataloging the particular nucleotide change, these signatures also take into account the preceding (5′) and following (3′) nucleotide, with a resulting 96 possible permutations for each nucleotide substitution (Alexandrov et al., 2013, 2020). Some of these mutational signatures are attributed to carcinogens and environmental exposures including some with known mechanisms for carcinogenesis. One such example is Signature 7, a result of UV activated cross-linking of pyrimidine (C & T) dimers. These DNA bases then require excision with a preferential replacement to thymine to correct the more commonly defective TT-dimer. Thus, thymine dimers are often replaced correctly, however any cytosine dimers involved by process are liable to undergo inappropriate replacement with CC > TT dinucleotide substitutions resulting at those sites (Figure 2A). Other mutational signatures are the result of ineffective cellular repair mechanisms. For instance, Signature 10 is the result of a specific mutation in DNA polymerase epsilon, a proof-reading domain of DNA polymerase. This specific mutation results in large numbers of mutations during DNA replication that are not corrected, preferentially C > A and C > T mutations on the leading strand (Figure 2B). Of note, tumors with DNA polymerase epsilon mutations also display a high TMB.

The list of exogenous and endogenous mutagens is long; however, not all mutational signatures described have a clearly defined mechanism of pathogenesis. Some signatures are clearly isolated within a small number of specific cancer types but the link to a specific etiology is unclear; whereas others are common across a wide spectrum of cancer groups (Helleday et al., 2014). It is likely that these currently uncertain mutational signatures will be eventually be tied to either environmental or cellular pathways of mutagenesis with future research and large-scale data analysis.

The determination of which mutational signature is present in a given tumor has multiple indications for clinical utility. In patients with complex medical histories and tumors of unknown origin, molecular signatures can assist in identifying the tumor type; for example, a patient with a significant smoking history with numerous cutaneous invasive squamous cell carcinomas and a new lung squamous cell carcinoma could have either a new lung primary or a solitary metastasis from the patient’s skin carcinoma. If that tumor contained a UV signature, the lung lesion could be confidently diagnosed as a metastasis and not a primary lung squamous cell carcinoma which would have significant differences in clinical management (Sholl et al., 2016). Mutational signatures may also be used to guide therapy selection, such as microsatellite instability in the case of ICI as well as homologous recombination deficiency in the setting of poly ADP ribose polymerase (PARP) inhibition, both of which will be detailed in later sections.

## Microsatellite Instability

Microsatellites are regions within the genome with repetitive nucleotide sequences. DNA polymerases have a difficult time replicating these regions and will slip during replication with resulting insertions or deletions of nucleotides. These insertions and deletions are typically recognized and repaired by the mismatch repair system (MMR). The MMR system is a complex of proteins including proteins MLH1, MSH2, MSH6, and PMS2, among others, that work to identify and replace base-pair incongruity. In situations with defective MMR, the uncorrected errors will remain and loci with microsatellites will show “instability” (MSI), new alleles appearing at those positions of varying lengths.

Defects in MMR can either be inherited or acquired. Inherited MMR deficiencies such as is seen in Lynch syndrome is not rare in cancer specimens with a prevalence of 0.4% in the general population (Haraldsdottir et al., 2017). The possibility of an inherited cancer predisposition syndrome has implications for cancer screening as well as for other family members and family planning and therefore MSI has been of clinical concern for many years. Patients with Lynch syndrome have up to an estimated 75% lifetime risk of developing colon cancer as well as increased risk for endometrial cancer, other gastrointestinal and genitourinary tract cancers, prostate, ovarian, breast and skin cancers (Hunter et al., 2017).

Tumors with MSI have traditionally been detected with a PCR assay that amplifies a select number of microsatellite loci with subsequent capillary electrophoresis to separate the size of fragments. Five loci were selected for clinical use in 1996, termed the “Bethesda panel” and formally endorsed a few years later by the National Cancer Institute Workshop on Microsatellite Instability for Cancer Detection and Familial Predisposition (Boland et al., 1998). These loci included two mononucleotide repeats (BAT25 and BAT26) and three dinucleotide repeats (D2S123, D5S346, and D17S250). The selection was based on the reproducibility to discriminate between microsatellite stable (MSS) and microsatellite instable (MSI) colorectal tumors. The applicability of these five loci to endometrial carcinoma, or other MSI non-colorectal carcinomas has been somewhat poor, with false negative results on PCR with gel electrophoresis (Wang et al., 2017). This is partially due a smaller nucleotide shift in fragment size variation. For instance, colorectal cancer has a mean nucleotide shift of six base pairs whereas endometrioid endometrial carcinoma has a mean shift of three base pairs.

Large-scale NGS panels often inherently sequence large numbers of microsatellites and can be designed to sequence additional loci, such as to include the five PCR loci among others. These hundreds of loci provide a global picture of microsatellite stability across the genome and therefore can be more sensitive for non-colorectal carcinomas with MSI (Hause et al., 2016). As in the PCR-based assay, NGS is inherently error-prone in these regions, and so microsatellites will have numerous different sized insertions/deletions which can be tabulated at each locus. Higher infidelity at these microsatellites is seen in tumors with microsatellite instability. Various bioinformatic approaches and algorithms have been developed to detect MSI using NGS data including mSINGS, MSIsensor, and MANTIS to name a few and have been reviewed in detail elsewhere (Yamamoto and Imai, 2019). The simultaneous sequencing of MMR proteins, such as *MLH1, MSH2*, and *MSH6*, can further corroborate some cases of MMR-deficient tumors, although no mutation will be seen in cases of hypermethylation of the *MLH1* promoter, a common mechanism of somatic acquisition of MMR deficiency (Baudrin et al., 2018), and *PMS2* is difficult to sequence due to extensive pseudogenes and homology throughout the genome.

Overall, NGS-based MSI testing has a higher diagnostic sensitivity than traditional PCR methods especially in cases of non-colorectal carcinomas (Baudrin et al., 2018). Additionally, because NGS testing is commonly performed on tumors that are not routinely screened with MMR immunohistochemistry, it has the ability to detect MSI in cancer types that would not normally be interrogated using traditional diagnostic algorithms. Finally, of note, tumors with MSI also have a high mutational burden (high TMB) and are more likely to express neoantigens that the immune system can identify as foreign and destroy. As such, MSI status was the Food and Drug Administration’s (FDA) first tumor-agnostic biomarker approval, with indications for ICI regardless of tumor type (Lemery et al., 2017; Marcus et al., 2019). This landmark approval was based on the knowledge that the biology of MSI was similar across tumor types and the overall response rate observed across five separate single arm clinical trials. The overall response rate was approximately 39.6% across 149 patients with 15 different tumor types and 78% of responses lasting at least 6 months (Le et al., 2015, 2017).

## Homologous Recombination Deficiency

Multiple DNA repair mechanisms exist for different types of DNA damage. The most reliable method for repairing double stranded DNA breaks is the homologous recombination pathway (Wright et al., 2018). Homologous recombination is a process with multiple steps: first, the double strand break must be recognized with protein binding. These bound proteins assist in the invasion of the damaged end to an intact homologous region for DNA synthesis. After DNA synthesis occurs, the complex repairs remaining nicks and disassembles. Numerous proteins are involved in these steps, including BRCA1, BRCA2, CHK2, NBN, and many others. When any of the proteins involved in homologous recombination are deficient (HRD), the deficient cells will not be able to repair double strand breaks through this mechanism. These cells then rely on alternate methods of repair, such as the error-prone method non-homologous end joining (NHEJ). In NHEJ two severed helixes fuse together so that DNA replication can proceed. This fusion is done without concern for fidelity of the DNA and results in translocations, insertions and deletions (Chang et al., 2017).

Poly ADP ribose polymerase inhibitors were initially discovered to elucidate exquisite sensitivity from *BRCA1/2* mutated cells (Bryant et al., 2005; Davies et al., 2017). This sensitivity is the result of the synthetic lethality of cells with HRD and concomitant PARP inhibition. Clinically, a defect in homologous recombination (HRD) suggests the patient will have a good response to PARP inhibitors as well as platinum chemotherapy (24, 26, 27). Platinum based chemotherapy induces DNA crosslinking. Cells attempt to repair the cross-linked DNA though nucleotide excision. The removal of nucleotides results in multiple single strand breaks (SSB). PARP is an enzyme that binds to SSB, excises a single base and then and recruits DNA repair enzymes to repair the strand including the break. In cells exposed to PARP inhibitors, SSB remain and, after replication, the SSB become double strand breaks. If the cell has intact homologous recombination, the cell can easily repair these double strand breaks and survive. In cells with a deficiency in homologous recombination however, the catastrophic number of double strand breaks cannot be appropriately repaired, and the cell undergoes apoptosis (Farmer et al., 2005). Three PARP inhibitors (olaparib, rucaparib, and niraparib) are currently approved by the FDA for ovarian cancer whereas olaparib and talazoparib are approved for breast cancer.

Sequencing of cancer specimens can identify whether there are mutations (either germline or somatic) in many of the genes involved in homologous recombination, such as a mutation in *BRCA1*. Additionally, mutational patterns exist that are also indicative of HRD, so-called “genomic scars” (Hoppe et al., 2018). Several different metrics have been proposed to identify this biomarker of DNA repair deficiency including Signature No. 3, HRDetect, deletions with microhomology, loss of heterozygosity (LOH), telomeric allelic imbalance (TAI), and large-scale state transitions (LST) (Alexandrov et al., 2013; Zhao et al., 2017). Many of these metrics are measures of HRD and a reliance on flawed alternate double-stranded break repair mechanisms (Abkevich et al., 2012). Signature No. 3 has been identified several different tumor types, including breast, ovarian, prostate, and gastric cancers (Alexandrov et al., 2013; Zhao et al., 2017). HRDetect has been shown to have utility in predicting “BRCA deficiency” as defined by either somatic or germline mutations in *BRCA1* as well as promoter hypermethylation in several tumor types such as breast, ovarian, and pancreatic cancers (Davies et al., 2017).

**FIGURE 3:**
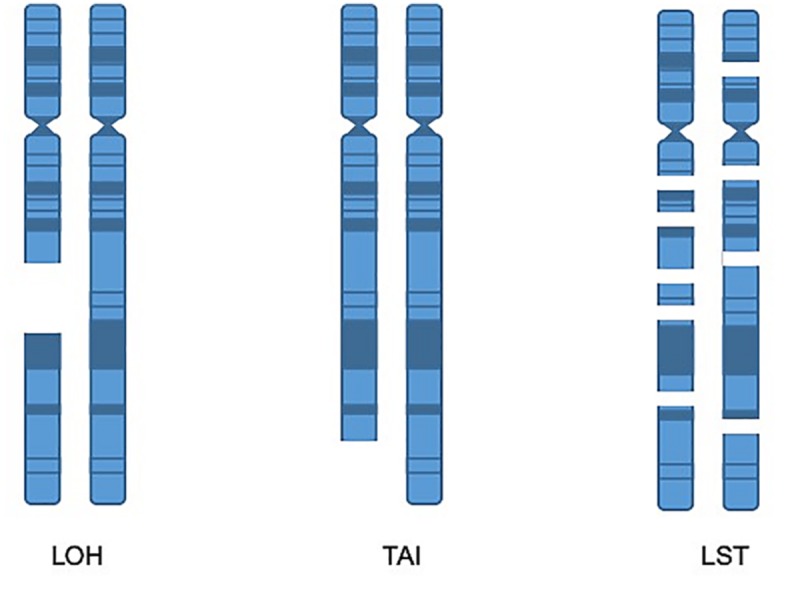
Homologous recombination deficiency metrics. LOH score is determined by the number of areas detected with loss of heterozygosity that are larger than 15 Mb, excluding entire chromosome loss. TAI is a metric accounting for chromosomal breaks that extend to the telomere. The LST score is a summation of the number of chromosomal breaks that are at least 10 Mb, but less than 15 Mb, with a distance between breaks of at least 3 Mb.

Although several of the HRD metrics were originally defined and identified through SNP array, NGS also has the capabilities to identify these metrics and WES is an excellent tool for capturing these biomarkers (Sztupinszki et al., 2018). One method for identifying tumors which harbor HRD is to obtain a score that is the sum total of the following metrics: LOH, LST, and TAI (Abkevich et al., 2012; Birkbak et al., 2012; Popova et al., 2012; Figure 3). While various combination scores have been proposed, the most data exists for using the combination of LOH, LST, and TAI and has been marketed by Myriad Genetics as the myChoice HRD test (Timms et al., 2014). Details of how these metrics are calculated are described in the original publications. In brief, the LOH score is determined by the number of areas detected with LOH that are larger than 15 Mb, excluding entire chromosome loss which is believed to be due to a different mechanism. The LST score is a summation of the number of chromosomal breaks that are at least 10 Mb large, but less than 15 Mb, and they must have a distance between breaks of 3 Mb to be counted as a separate break. Finally, the TAI is a simple accounting of any break that extends to the telomere. In a multivariable model, the combination score of LOH, TAI, and LST was significantly associated with *BRCA* deficiency in breast cancer specimens. Additionally, the combined score was also retrospectively validated to predict response to neoadjuvant platinum therapy in triple-negative breast cancer (TNBC) (64). The combined score was also shown to predict response in TNBC after chemotherapy regardless of BRCA mutation status (65). The myChoice HRD test was evaluated in the NOVA study of niraparib in ovarian cancer which showed a significant benefit in positive patients that was greater than the benefit noted for non-HRD patients (17). High HRD score (>42) and/or BRCA1/2 mutation is also associated with durable responses to olaparib (46). In contrast, among single score assays, only the Foundation Medicine LOH score has been validated prospectively in the phase III ARIEL 3 study (Coleman et al., 2017).

While HRD biomarkers are very promising, they are not without limitations. For example, combined HRD score was not able to predict platinum sensitivity in the TNT study evaluating metastatic TNBC (Tutt et al., 2018). There are several possible explanations for the failure of this biomarker in this setting and in others, such as the previous exposure to chemotherapy leading to the emergence of resistance mechanisms, which is supported by data suggesting that HRD genomic scars have improved performance in neoadjuvant settings (Telli et al., 2016). Described mechanisms of resistance include the acquisition of “reversion” mutations that can restore functional homologous recombination in the tumor and yet the genomic scars remain. Such reversion mutations have been reported in *BRCA1/2*, *PALB2*, and *RAD51C/D* (Edwards et al., 2008; Sakai et al., 2008; Goodall et al., 2017; Kondrashova et al., 2017). An additional mechanism of resistance which may be unrelated to HRD and undetectable by any of the related markers is the increased expression of the efflux transporter ATP-binding cassette transporter B1 (ABCB1; P-glycoprotein; multidrug resistance protein 1) (Rottenberg et al., 2008). Further data is needed to fully realize the clinical utility of the various HRD biomarker assays.

## Cancer Mutational Patterns: Current and Future Clinical Implementation in Cancer Precision Medicine

Somatic cancer sequencing as performed by NGS is an excellent, comprehensive test for identifying simple mutations, complex genetic alterations, quantifying TMB, determining MSI and HRD and recognizing potential mutational signatures. The results derived from patterns seen in NGS data are actionable: both MSI and HRD have FDA-approved indications for therapy, and TMB is also an emerging biomarker for immunotherapy response.

Separately, some results are not directly actionable but do have clinical relevance: mutational signatures assist in the diagnostic challenges that result from limitations in traditional histology and immunohistochemistry (Sholl et al., 2016). Mutational signatures suggest underlying mechanisms in disease and causative environmental mutagens which may point to a particular diagnosis in particular cases. However, challenges remain for implementing NGS as a comprehensive test for TMB, MSI, mutational signatures and HRD in addition to identification of traditional genetic alterations. NGS, although substantially more affordable in recent years, is still an expensive test. From a laboratory development and technical standpoint there are also significant issues, including a lack of standardization for calculating many of the metrics, difficulty in obtaining validation samples with known “ground truth,” and lack of matched normal testing in many laboratories. Even if several of these obstacles are overcome, many of the NGS-based metrics are computationally complex and require extensive bioinformatics development and deployment, which can be a significant bottleneck and hurdle for many laboratories.

In this age of “personalized medicine” no single result will determine a patient’s fate or optimal treatment, instead clinicians must synthesize huge numbers of complex test results into a single coherent clinical plan. The vast quantity of data produced from primary and secondary analysis of large NGS panel results makes this single test an increasingly attractive option for integrated reporting.

## Author Contributions

LR and MP contributed to the content, writing, editing, and review of the manuscript.

## Conflict of Interest

The authors declare that the research was conducted in the absence of any commercial or financial relationships that could be construed as a potential conflict of interest.
